# Excess mortality in US Veterans during the COVID-19 pandemic: an individual-level cohort study

**DOI:** 10.1093/ije/dyad136

**Published:** 2023-10-06

**Authors:** Daniel M Weinberger, Krishnan Bhaskaran, Caroline Korves, Brian P Lucas, Jesse A Columbo, Anita Vashi, Louise Davies, Amy C Justice, Christopher T Rentsch

**Affiliations:** Department of Epidemiology of Microbial Diseases, Yale School of Public Health, New Haven, CT, USA; Center for Interdisciplinary Research on AIDS, Yale School of Public Health, New Haven, CT, USA; Faculty of Epidemiology and Population Health, London School of Hygiene & Tropical Medicine, London, UK; Department of Veterans Affairs Medical Center, Clinical Epidemiology Program, White River Junction, VT, USA; Department of Veterans Affairs Medical Center, VA Outcomes Group, White River Junction, VT, USA; The Dartmouth Institute for Health Policy & Clinical Practice, Geisel School of Medicine at Dartmouth, Hanover, NH, USA; Department of Veterans Affairs Medical Center, VA Outcomes Group, White River Junction, VT, USA; The Dartmouth Institute for Health Policy & Clinical Practice, Geisel School of Medicine at Dartmouth, Hanover, NH, USA; Section of Vascular Surgery, Dartmouth Hitchcock Medical Center, Lebanon, NH, USA; Center for Innovation to Implementation, VA Palo Alto Health Care System, Menlo Park, CA, USA; Department of Emergency Medicine, University of California, San Francisco, CA, USA; Department of Veterans Affairs Medical Center, VA Outcomes Group, White River Junction, VT, USA; The Dartmouth Institute for Health Policy & Clinical Practice, Geisel School of Medicine at Dartmouth, Hanover, NH, USA; Department of Surgery—Otolaryngology Head & Neck Surgery, Geisel School of Medicine at Dartmouth, Hanover, NH, USA; Center for Interdisciplinary Research on AIDS, Yale School of Public Health, New Haven, CT, USA; Department of Internal Medicine, Yale School of Medicine, New Haven, CT, USA; Department of Veterans Affairs, VA Connecticut Healthcare System, West Haven, CT, USA; Faculty of Epidemiology and Population Health, London School of Hygiene & Tropical Medicine, London, UK; Department of Internal Medicine, Yale School of Medicine, New Haven, CT, USA; Department of Veterans Affairs, VA Connecticut Healthcare System, West Haven, CT, USA

**Keywords:** COVID-19, excess mortality, electronic health records, frailty, comorbidity, Veterans

## Abstract

**Background:**

Most analyses of excess mortality during the COVID-19 pandemic have employed aggregate data. Individual-level data from the largest integrated healthcare system in the US may enhance understanding of excess mortality.

**Methods:**

We performed an observational cohort study following patients receiving care from the Department of Veterans Affairs (VA) between 1 March 2018 and 28 February 2022. We estimated excess mortality on an absolute scale (i.e. excess mortality rates, number of excess deaths) and a relative scale by measuring the hazard ratio (HR) for mortality comparing pandemic and pre-pandemic periods, overall and within demographic and clinical subgroups. Comorbidity burden and frailty were measured using the Charlson Comorbidity Index and Veterans Aging Cohort Study Index, respectively.

**Results:**

Of 5 905 747 patients, the median age was 65.8 years and 91% were men. Overall, the excess mortality rate was 10.0 deaths/1000 person-years (PY), with a total of 103 164 excess deaths and pandemic HR of 1.25 (95% CI 1.25–1.26). Excess mortality rates were highest among the most frail patients (52.0/1000 PY) and those with the highest comorbidity burden (16.3/1000 PY). However, the largest relative mortality increases were observed among the least frail (HR 1.31, 95% CI 1.30–1.32) and those with the lowest comorbidity burden (HR 1.44, 95% CI 1.43–1.46).

**Conclusions:**

Individual-level data offered crucial clinical and operational insights into US excess mortality patterns during the COVID-19 pandemic. Notable differences emerged among clinical risk groups, emphasizing the need for reporting excess mortality in both absolute and relative terms to inform resource allocation in future outbreaks.

Key Messages

- Most analyses of excess mortality during the COVID-19 pandemic have focused on evaluations of aggregate data, which may miss important individual-level drivers of excess mortality that may serve as future targets for improvement initiatives.
- Using individual-level data from a national integrated healthcare system, we estimated absolute and relative excess mortality and number of excess deaths overall and within demographic and clinical subgroups.
- Absolute rates of excess mortality were typically highest in groups in which the baseline rate of mortality was higher, namely in older age groups and among those with more comorbidities and higher levels of physiologic frailty.
- Relative measures of excess mortality were typically greatest among younger age groups and among those with lower physiologic frailty and fewer comorbidities.
- Relative measures of excess mortality attenuated but remained elevated after censoring follow-up at first documented SARS-CoV-2 infection or COVID-19, suggesting that factors beyond SARS-CoV-2 infection contributed to the observed excess mortality during the pandemic.

## Introduction

During the COVID-19 pandemic, there was a substantial increase in rates of death due to any cause.^1–3^ Rates of deaths that exceed expected levels are referred to as excess deaths, which were observed globally.^4–6^ Some geographic regions, risk groups and age groups experienced larger excesses,^4^^,^^6^ namely of which were directly attributed to the virus, particularly in older adults.^7^ Other evidence points to healthcare system-level factors, such as disruptions to healthcare system function, personal health management and healthcare utilization.^8^^,^^9^ However, the pandemic also caused major disruptions in society, possibly contributing to overdoses,^10^ suicides^8^ or violent crime.^11^ The risk of death due to COVID-19 as well as susceptibility to these secondary effects of the pandemic depends on a complex set of factors including the underlying health status of an individual.

To improve understanding of the drivers of excess deaths during the COVID-19 pandemic, including those caused directly by the virus and those indirectly caused by pandemic disruptions, it is necessary to consider detailed individual-level characteristics. Most analyses of excess deaths during the COVID-19 pandemic focused on evaluations of aggregate data, looking at changes in numbers of deaths compared with a pre-pandemic baseline. Linking these time series with data on other characteristics and risk factors can provide a broader understanding of the drivers of excess deaths.^5^^,^^7^ However, even this strategy may miss important individual-level drivers of excess mortality that may serve as future targets for improvement initiatives. With individual-level data from an integrated care system, it is possible to address this gap in knowledge and obtain a better understanding of the individual demographic and clinical factors that influence excess mortality and to identify the patient subgroups that experienced the greatest burden of excess deaths. Using individual-level data from the largest integrated healthcare system in the US, we estimated excess mortality rates and number of deaths overall and within demographic, comorbidity and physiologic frailty subgroups. These analyses provide a more comprehensive picture of excess mortality than can be obtained from aggregate data alone.

## Methods

### Data source

The US Department of Veterans Affairs (VA) serves 9 million Veterans annually at 171 medical centres and 1112 outpatient sites nationwide.^12^ All care is recorded in an electronic health record with daily uploads into the VA Corporate Data Warehouse. Available data include demographics, outpatient and inpatient encounters, diagnoses, laboratory measures and death records.

This study was approved by the institutional review boards of VA Connecticut Healthcare System and Yale University. It has been granted a waiver of informed consent and is Health Insurance Portability and Accountability Act compliant. This study is reported as per the Strengthening the Reporting of Observational Studies in Epidemiology (STROBE) and reporting of studies conducted using observational routinely collected health data (RECORD) guidelines (see Supplementary material, available as Supplementary data at *IJE* online).

### Study design and population

We conducted an observational cohort study including all Veterans aged ≥18 years in active care in the VA between 1 March 2018 and 28 February 2022. We allowed for 2 years of pre-pandemic follow-up (i.e. 1 March 2018 to 29 February 2020) and 2 years of pandemic follow-up (i.e. 1 March 2020 to 28 February 2022), covering the same periods of the years to mitigate seasonal variation in mortality trends. Active VA care was defined as the presence of an outpatient or inpatient diagnostic code in the 2 years prior to each time period, in line with our previous work.^13^ The baseline date was defined as the latest of 1 March 2018 or 1 year after their first diagnosis code in the 2-year period before 1 March 2018, to allow for the recording of baseline covariates. Deaths were ascertained using inpatient records and VA death registry data to capture deaths outside of hospitalization. Patients were followed until the earliest of date of death, dropped out of care (i.e. 18 months after their last visit) or end of study (i.e. 28 February 2022).

### Covariates

We selected demographic and clinical characteristics that have been evaluated in prior reports as contributors to COVID-19 excess mortality in addition to validated measures of physiologic frailty and comorbidity burden. Demographics included age, sex, race/ethnicity, US census region (i.e. West, South, Midwest and Northeast) and residence type (i.e. urban, rural). Race and ethnicity were self-reported and categorized as White, Black, Hispanic or Latino (Hispanic), Asian, American Indian/Alaska Native, Pacific Islander/Native Hawaiian and people of mixed race. In line with previous work,^14^ patients who reported Hispanic ethnicity were included in the Hispanic group regardless of any other self-reported race. Residence type was defined using geographic information system coding based upon established criteria.^15^

The Veterans Aging Cohort Study Index (VACS Index) assesses physiologic frailty by calculating a summary score using a validated algorithm incorporating haemoglobin, alanine transaminase, aspartate transaminase, platelets, creatinine, hepatitis C status, albumin, white blood cell count, body mass index and age.^16^ The VACS Index is a validated and generalizable risk index that has been shown to predict and discriminate risk of morbidity and mortality in multiple settings.^17^^,^^18^ The Charlson Comorbidity Index (CCI) has been a mainstay measure of overall comorbidity burden for decades and is based on diagnostic codes across 17 clinical domains.^19^^,^^20^ We examined CCI as a summary score based on established methods as well as individual components in subgroup analyses. Both the VACS Index and the CCI were ascertained using the most recent laboratory measures and all diagnostic codes that were recorded in the 2 years prior to baseline, and the status was time-updated on 1 March 2020 using data from the 2 years prior to 1 March 2020. Patients could therefore be categorized in more than one CCI domain and these classifications may differ between the pre-pandemic and pandemic periods.

### Statistical analysis

First, we estimated the hazard of mortality during the pandemic period relative to the pre-pandemic period, adjusting for individual-level characteristics. We fit a Cox proportional hazards model with age as the underlying timescale and the main pandemic exposure variable defined as a time-updated covariate taking the value ‘0’ before 1 March 2020 and ‘1’ from 1 March 2020. Cox models estimated excess mortality adjusting the baseline hazard for (i) age only and (ii) additionally adjusting for demographic characteristics, VACS Index and CCI. Only race/ethnicity (5%) and VACS Index (26%) suffered from missing data. We included a missing category for these covariates under the assumption that associations between fully observed covariates and calendar time did not differ across missingness patterns, which would result in unbiased estimates.^21^^,^^22^ More details, including the specification of the fully adjusted Cox model, can be found in the Methods section in the Supplementary material (available as Supplementary data at *IJE* online).

In subgroup analyses, we estimated excess mortality rates, number of excess deaths and pandemic hazard ratios (HRs) measuring relative increases in mortality comparing pandemic to pre-pandemic follow-up within each demographic and clinical characteristic. Cox models were specified as the model described above but with the addition of an interaction term between the pandemic time binary indicator and the given characteristic. A separate model was fitted for each characteristic.

In secondary analyses, we repeated the analysis above for each of the 17 clinical domains of the CCI by fitting a separate Cox model with an interaction between the pandemic time variable and a binary indicator denoting the presence or absence of a diagnostic code within the relevant clinical domain. These models were not adjusted for CCI summary score to mitigate potential issues with collinearity between the CCI summary score and its individual components. Finally, we reran all models, censoring patients at the date of first recorded SARS-CoV-2 infection or COVID-19 diagnosis to understand the extent to which excess mortality could be attributed to COVID-19 vs all other causes. We used the national VA COVID-19 Shared Data Resource, encompassing verified data on all VA patients who had received a laboratory-confirmed diagnosis of SARS-CoV-2 infection as well as cases that were tested externally to the VA with a VA clinical note substantiating the diagnosis. We used Microsoft SQL Server Management Studio v18.11 for data management and SAS Enterprise Guide version 8.3 (SAS Institute, Cary, NC) for statistical analyses.

### Post-hoc analysis

In a post-hoc analysis, we split the pandemic follow-up time by individual-level vaccination status. We considered 14 days after receipt of a complete vaccination schedule (one or two doses, depending on product) as fully vaccinated and stratified follow-up by this date.

## Results

### Population characteristics

A total of 6 252 992 Veterans were alive and enrolled in the VA at the start of the study period. Of these, 347 245 were not active in care, resulting in 5 905 747 patients who were eligible for follow-up during the pre-pandemic period. Most patients (*n* = 5 488 957; 92.9%) continued follow-up during the pandemic period, whereas 416 790 (7.1%) dropped out of care or died during the pre-pandemic period. Of the 5 905 747 patients followed in the pre-pandemic period, the median age was 65.8 years (interquartile range 51.0–72.9), 91.4% were men, 68.1% were White, 17.1% were Black and 6.4% were Hispanic (Table 1). Patients were predominately (44.3%) located in the South and 65.0% resided in urban settings. Over half (53.9%) of all patients were categorized in the lowest (i.e. least frail) quartile of VACS Index, whereas 3.0% were in the highest quartile (i.e. most frail). Similarly, half (50.3%) of all patients had a CCI score of 0 indicating no recorded diagnoses across the 17 clinical domains, whereas 5.3% had a CCI score of ≥5. In order of decreasing prevalence, 24.6% were diagnosed with diabetes (uncomplicated), 15.1% were diagnosed with chronic obstructive pulmonary disease and 10.4% were diagnosed with diabetes (end-organ damage). Similar distributions of patient characteristics were observed among those followed during the pandemic period (Table 1).

**Table 1.** Population characteristics

Characteristic	In care during pre-pandemic period		In care during pandemic period	
	*n*	Column %	*n*	Column %
Number in care	5 905 747	100.0	5 488 957	100.0
Age (years)				
18–44	1 068 852	18.1	948 482	17.3
45–64	1 779 684	30.1	1 598 729	29.1
65–74	1 856 101	31.4	1 711 804	31.2
75–84	775 289	13.1	810 035	14.8
≥85	425 821	7.2	419 907	7.7
Sex				
Women	505 725	8.6	492 389	9.0
Men	5 400 022	91.4	4 996 568	91.0
Race/ethnicity				
White	4 019 281	68.1	3 708 945	67.6
Black	1 010 252	17.1	960 865	17.5
Hispanic	380 526	6.4	362 963	6.6
Asian	62 005	1.0	59 505	1.1
AI/AN	40 068	0.7	37 613	0.7
PI/NH	43 242	0.7	40 617	0.7
Mixed race	45 947	0.8	43 364	0.8
Missing	304 426	5.2	275 085	5.0
Region				
West	1 250 453	21.2	1 164 092	21.2
South	2 614 775	44.3	2 449 788	44.6
Midwest	1 276 192	21.6	1 176 417	21.4
Northeast	764 327	12.9	698 660	12.7
Residence type				
Rural	2 065 668	35.0	1 917 742	34.9
Urban	3 840 079	65.0	3 571 215	65.1
VACS Index^a^				
First quartile	3 185 621	53.9	2 886 589	52.6
Second quartile	670 522	11.4	676 597	12.3
Third quartile	331 541	5.6	329 386	6.0
Fourth quartile	178 877	3.0	174 874	3.2
Missing	1 539 186	26.1	1 421 511	25.9
Charlson Comorbidity Index^b^				
0	2 969 223	50.3	2 654 910	48.4
1	1 202 465	20.4	1 087 001	19.8
2	785 898	13.3	750 777	13.7
3	395 732	6.7	394 276	7.2
4	237 171	4.0	250 466	4.6
≥5	315 258	5.3	351 527	6.4
Myocardial infarction	114 967	1.9	120 485	2.2
Congestive heart failure	314 548	5.3	328 581	6.0
Peripheral vascular disease	397 563	6.7	413 310	7.5
Cerebrovascular accident	328 897	5.6	337 496	6.1
Hemiplegia	34 837	0.6	34 246	0.6
Dementia	150 975	2.6	145 257	2.6
COPD	890 890	15.1	874 682	15.9
Connective tissue disease	79 307	1.3	80 422	1.5
Peptic ulcer disease	38 404	0.7	37 194	0.7
Diabetes, uncomplicated	1 452 090	24.6	1 402 971	25.6
Diabetes, end-organ damage	615 266	10.4	634 723	11.6
Moderate to severe CKD	450 381	7.6	494 276	9.0
Liver disease, mild	301 840	5.1	296 213	5.4
Liver disease, moderate to severe	21 906	0.4	21 905	0.4
HIV/AIDS	26 684	0.5	25 833	0.5
Cancer, localized	421 098	7.1	435 252	7.9
Cancer, metastatic	33 826	0.6	36 594	0.7

AI/AN, American Indian/Alaska Native; CKD, chronic kidney disease; COPD, chronic obstructive pulmonary disorder; PI/NH, Pacific Islander/Native Hawaiian; VACS, Veterans Aging Cohort Study.

a The VACS Index assesses physiologic frailty using a previously validated algorithm. VACS Index quartiles were as follows: first (29.0–75.7), second (75.8–84.4), third (84.5–93.2) and fourth (93.3–157.9).

b The Charlson Comorbidity Index is a measure of overall comorbidity burden based on diagnostic codes across 17 clinical domains.

### Excess mortality

There were 358 664 recorded deaths among patients followed for 11 337 771 person-years (PY) during the pre-pandemic period and 429 289 recorded deaths among those followed for 10 309 181 PY during the pandemic period, resulting in mortality rates of 31.6 and 41.6 deaths/1000 PY, respectively. Overall, the excess mortality rate was 10.0 deaths/1000 PY, resulting in a total of 103 164 excess deaths. Adjusting for age only, the rate of deaths during the pandemic period was 27% higher (95% CI 26%–27%; Table 2) than in the pre-pandemic period (‘excess mortality’). Excess mortality was slightly lower at 25% (95% CI 25%–26%) after additionally adjusting for demographic characteristics, physiologic frailty and comorbidity burden.

**Table 2.** Excess mortality estimates adjusted for age and demographic and clinical characteristics, with and without censoring of COVID-19 follow-up

Characteristic	Age-adjusted HR (95% CI)	Fully adjusted HR (95% CI)	Censoring COVID-19 follow-up HR (95% CI)
Period			
Pre-pandemic	1.00 (ref)	1.00 (ref)	1.00 (ref)
Pandemic	1.27 (1.26–1.27)	1.25 (1.25–1.26)	1.19 (1.19–1.20)
Sex			
Men		1.00 (ref)	1.00 (ref)
Women		0.65 (0.64–0.66)	0.65 (0.65–0.67)
Race/ethnicity			
White		1.00 (ref)	1.00 (ref)
Black		0.86 (0.85–0.87)	0.85 (0.84–0.86)
Hispanic		0.75 (0.75–0.76)	0.74 (0.73–0.75)
Asian		0.62 (0.61–0.65)	0.62 (0.60–0.64)
AI/AN		1.11 (1.08–1.14)	1.09 (1.06–1.13)
PI/NH		0.91 (0.89–0.94)	0.90 (0.87–0.93)
Mixed race		0.96 (0.94–0.99)	0.96 (0.93–0.99)
Missing		1.07 (1.06–1.08)	1.08 (1.07–1.09)
Region			
South		1.00 (ref)	1.00 (ref)
Midwest		0.96 (0.96–0.97)	0.96 (0.96–0.97)
Northeast		0.93 (0.93–0.94)	0.93 (0.92–0.94)
West		1.01 (1.00–1.01)	1.00 (1.00–1.01)
Residence type			
Rural		1.00 (ref)	1.00 (ref)
Urban		0.98 (0.98–0.99)	0.98 (0.98–0.99)
VACS Index^a^			
First quartile		1.00 (ref)	1.00 (ref)
Second quartile		1.93 (1.91–1.95)	1.95 (1.93–1.97)
Third quartile		2.96 (2.93–2.98)	3.01 (2.98–3.03)
Fourth quartile		4.95 (4.91–5.00)	5.06 (5.01–5.10)
Missing		2.23 (2.21–2.25)	2.28 (2.26–2.30)
Charlson Comorbidity Index^b^			
0		1.00 (ref)	1.00 (ref)
1		1.63 (1.62–1.65)	1.63 (1.62–1.64)
2		1.84 (1.83–1.85)	1.83 (1.81–1.84)
3		2.36 (2.34–2.38)	2.34 (2.32–2.36)
4		2.57 (2.55–2.59)	2.54 (2.52–2.57)
≥5		3.87 (3.84–3.90)	3.82 (3.79–3.85)

ref, referent category; AI/AN, American Indian/Alaska Native; COVID-19, coronavirus disease 2019; HR, hazard ratio; PI/NH, Pacific Islander/Native Hawaiian; VACS, Veterans Aging Cohort Study.

a The VACS Index assesses physiologic frailty using a previously validated algorithm. VACS Index quartiles were as follows: first (29.0–75.7), second (75.8–84.4), third (84.5–93.2) and fourth (93.3–157.9).

b The Charlson Comorbidity Index is a measure of overall comorbidity burden based on diagnostic codes across 17 clinical domains.

### Subgroup analyses

**Figure 1.**
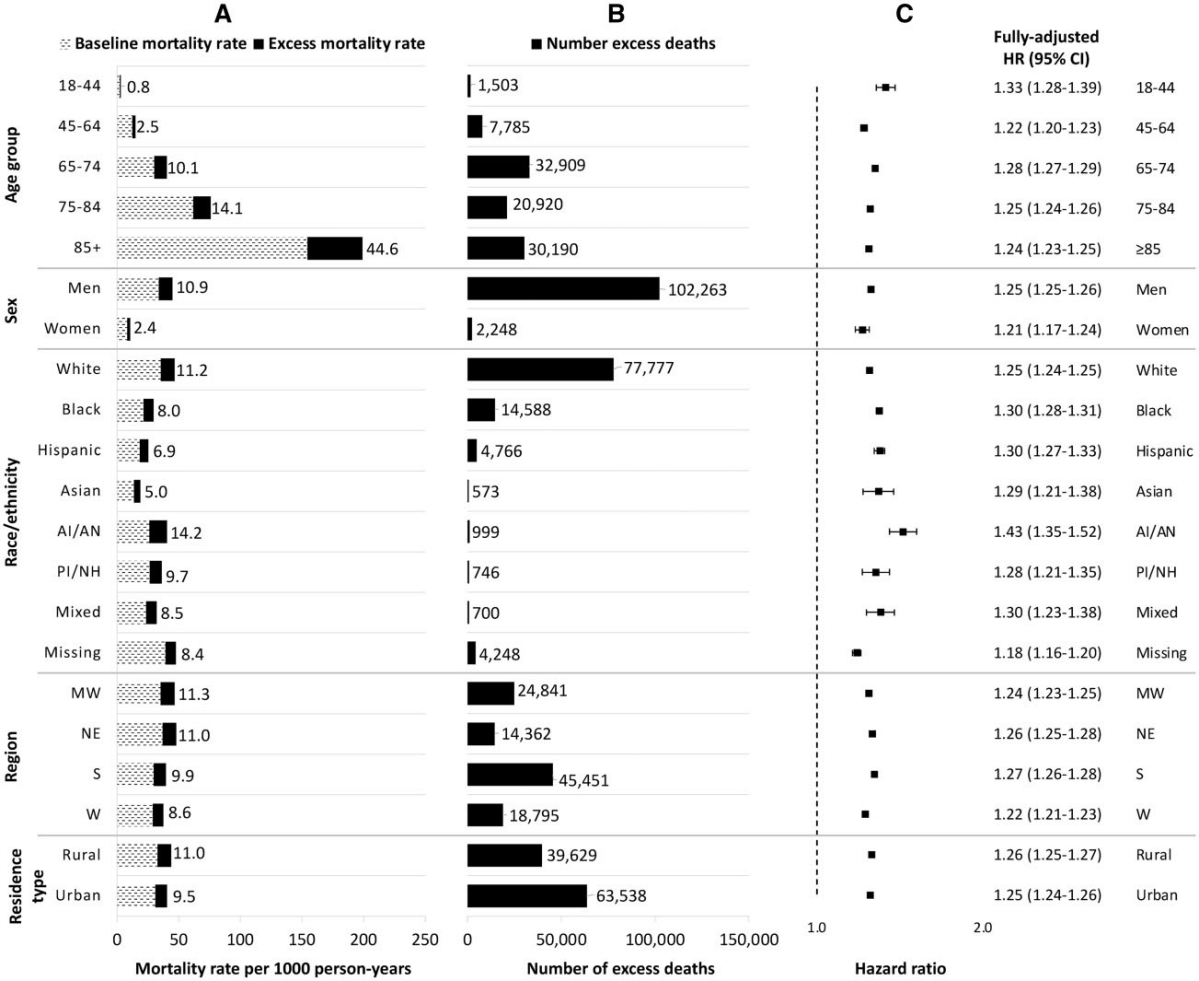
Mortality rates, number of excess deaths and hazard ratios comparing pre-pandemic and pandemic mortality by demographic subgroup. The numbers of excess deaths are adjusted for the characteristic of interest only. Fully adjusted hazard ratios were derived from a separate Cox model for each characteristic with an interaction between the pandemic time variable and each given characteristic, and adjusted for all demographics, physiologic frailty and comorbidity burden. In (A), the numbers listed refer to the excess mortality rates. HR, hazard ratio; AI/AN, American Indian/Alaska Native; PI/NH, Pacific Islander/Native Hawaiian; MW, Midwest; NE, Northeast; S, South; W, West

By age group, the rate of excess deaths was highest among patients aged ≥85 years (44.6 deaths/1000 PY) and the absolute number of excess deaths was highest among patients aged 65–74 years (32 909 excess deaths). The relative increase in the hazard of death was highest among patients aged 18–44 years (HR 1.33, 95% CI 1.28–1.39), though this group experienced the lowest absolute number of excess deaths (Figure 1 and Supplementary Table S1, available as Supplementary data at *IJE* online). By race and ethnicity, although the absolute number of excess deaths was greatest among White patients (77 777 excess deaths), the excess mortality rate (14.2 deaths/1000 PY) and pandemic hazard ratio (HR 1.43, 95% CI 1.35–1.52) were highest among American Indian/Alaska Native patients.

**Figure 2.**
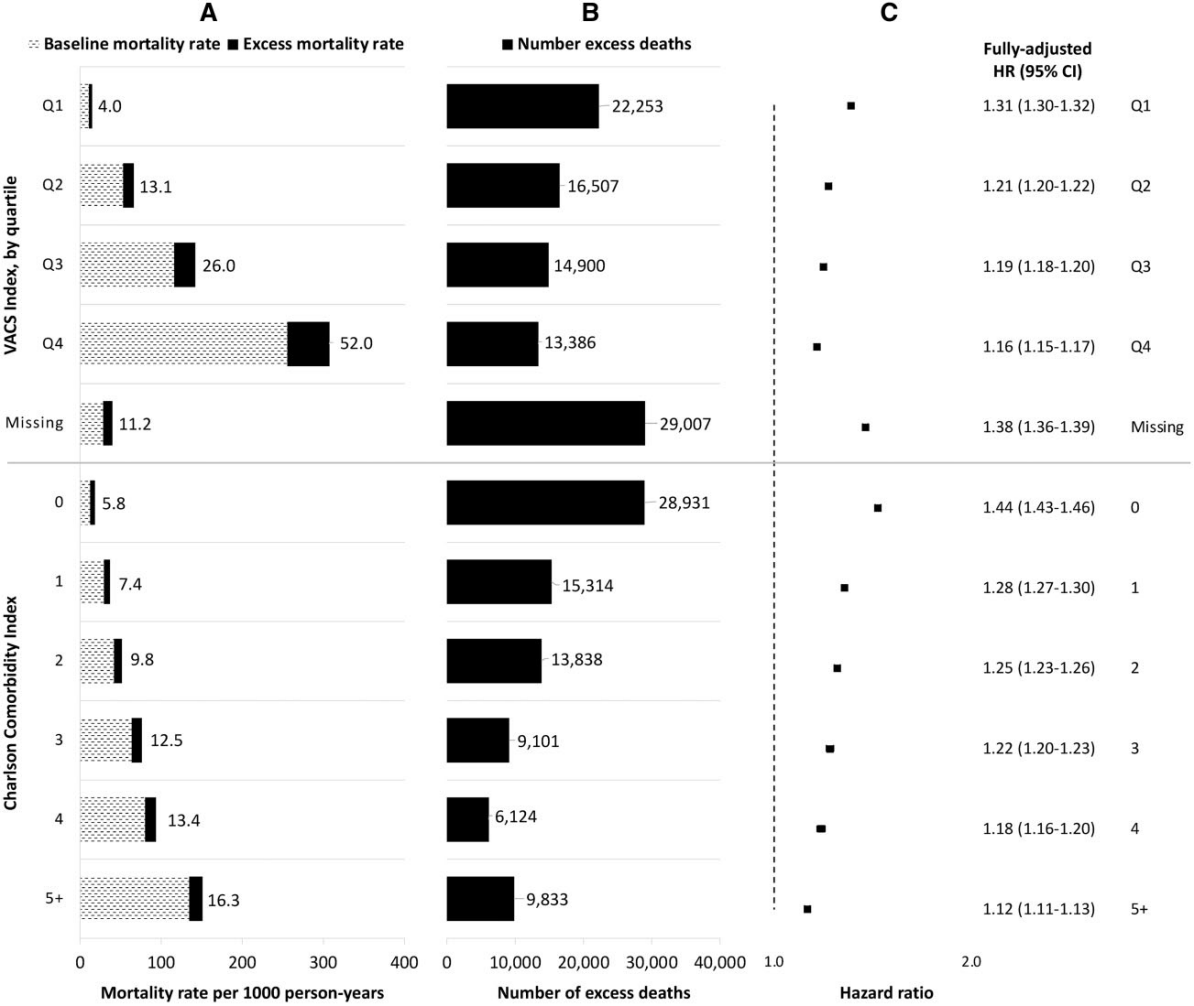
Mortality rates, number of excess deaths and hazard ratios comparing pre-pandemic and pandemic mortality by physiologic frailty and comorbidity burden. The numbers of excess deaths are adjusted for the characteristic of interest only. Fully adjusted hazard ratios were derived from a separate Cox model for each characteristic with an interaction between the pandemic time variable and each given characteristic, and adjusted for all demographics, physiologic frailty and comorbidity burden. In (A), the numbers listed refer to the excess mortality rates. HR, hazard ratio; VACS, Veterans Aging Cohort Study; Q1, first quartile (29.0–75.7); Q2, second quartile (75.8–84.4); Q3, third quartile (84.5–93.2); Q4, fourth quartile (93.3–157.9); CCI, Charlson Comorbidity Index

The excess mortality rate (52.0 deaths/1000 PY) was highest among the most frail patients (fourth quartile of physiologic frailty as measured by the VACS Index), whereas the highest absolute number of excess deaths (22 253 excess deaths) and largest relative increase in mortality (HR 1.31, 95% CI 1.30–1.32) was observed among the least frail patients (Figure 2). Similarly, whereas the excess mortality rate (16.3 deaths/1000 PY) was highest among patients with the highest comorbidity burden (CCI score ≥5), the highest absolute number of excess deaths (28 931 excess deaths) and relative increase in mortality (HR 1.44, 95% CI 1.43–1.46) were observed among patients with the lowest comorbidity burden (CCI score of 0).

### Secondary analyses

**Figure 3.**
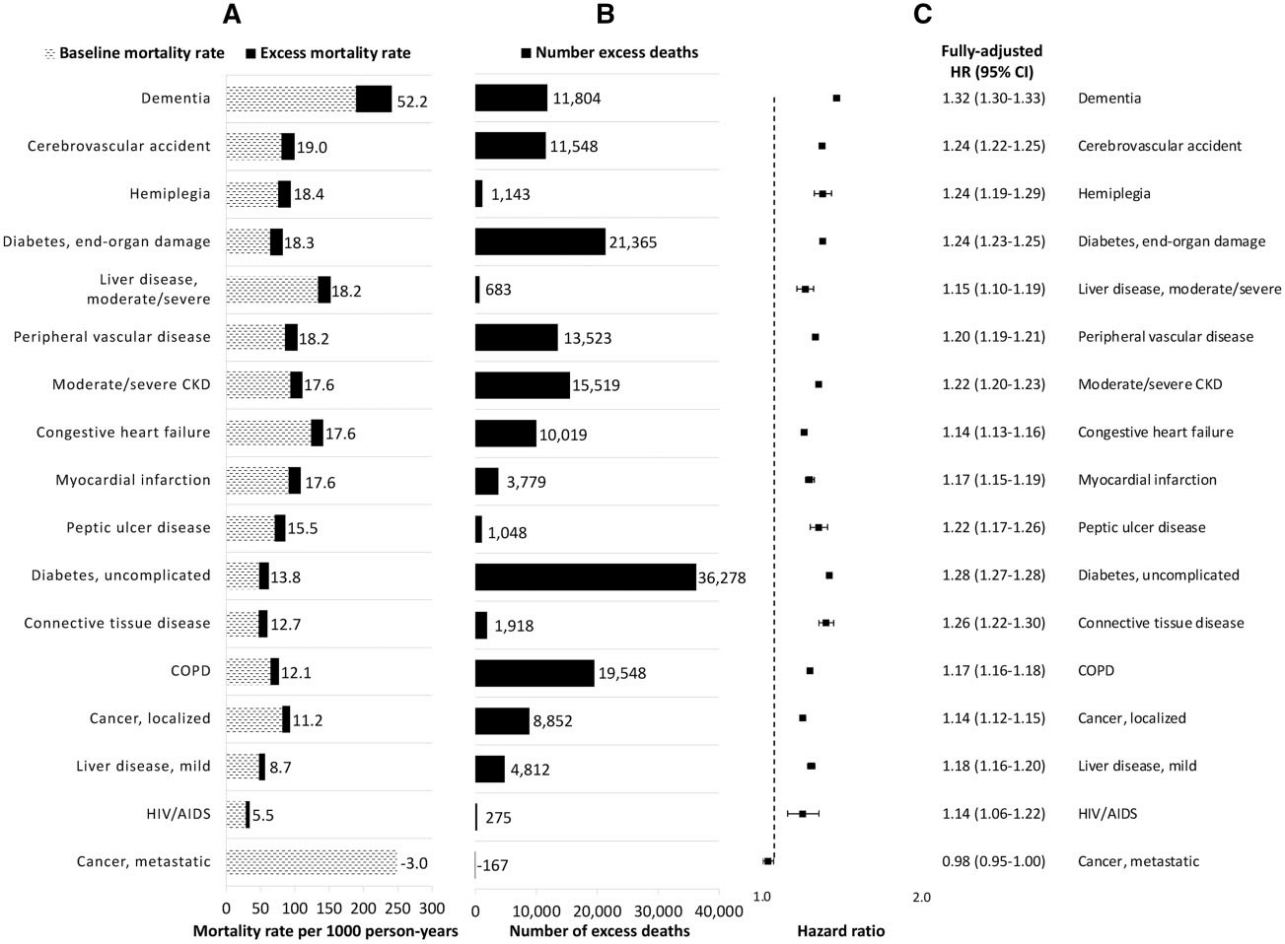
Mortality rates, number of excess deaths and hazard ratios comparing pre-pandemic and pandemic mortality by clinical domain (ordered by decreasing excess mortality rate). The numbers of excess deaths are adjusted for the characteristic of interest only. Patients can contribute to more than one clinical domain. Fully adjusted hazard ratios were derived from a separate Cox model for each clinical domain with an interaction between the pandemic time variable and a binary indicator denoting the presence or absence of a diagnostic code within the relevant clinical domain, and adjusted for all demographic characteristics and physiologic frailty. In (A), the numbers listed refer to the excess mortality rates. HR, hazard ratio; CKD, chronic kidney disease; COPD, chronic obstructive pulmonary disease

Patients with dementia had the highest excess mortality rate (52.2 deaths/1000 PY) and highest relative increase in mortality (HR 1.32, 95% CI 1.30–1.33; Figure 3 and Supplementary Table S2, available as Supplementary data at *IJE* online). However, patients with diabetes had the highest number of excess deaths, including 36 278 excess deaths among those with uncomplicated diabetes and 21 365 excess deaths among those with diabetes-associated end-organ damage. There were 626 973 (11.4%) patients who had evidence of SARS-CoV-2/COVID-19 during the first 2 years of the pandemic. After censoring COVID-19 follow-up, the pandemic HR attenuated from 1.25 (95% CI 1.25–1.26) to 1.19 (95% CI 1.19–1.20) (Table 1). Changes in the pandemic HR after censoring COVID-19 follow-up followed a similar pattern for all demographic and clinical subgroups, with the largest absolute differences observed among Hispanic patients (HR 1.30, 95% CI 1.27–1.33 before censoring and HR 1.19, 95% CI 1.17–1.22 after censoring) and those with the lowest VACS Index (HR 1.31, 95% CI 1.30–1.32 before censoring and HR 1.21, 95% CI 1.20–1.22 after censoring; Supplementary Table S1, available as Supplementary data at *IJE* online).

### Post-hoc analysis

A total of 2 999 915 (54.7%) of 5 488 957 patients in care at the start of the pandemic were fully vaccinated during the study period. After stratifying by individual-level vaccination status, excess mortality was 39% (95% CI 39%–40%) when comparing unvaccinated pandemic follow-up time with pre-pandemic time (Supplementary Table S3, available as Supplementary data at *IJE* online). Patterns of excess mortality reversed when comparing vaccinated pandemic follow-up time to pre-pandemic time (HR 0.89, 95% CI 0.88–0.89) or to unvaccinated pandemic follow-up time (HR 0.64, 95% CI 0.63–0.64).

## Discussion

The impact of the COVID-19 pandemic on overall rates of mortality has been well documented; however, previous work has largely relied on aggregate population-level data. Using individual-level electronic health record data from the largest integrated healthcare system in the US, we demonstrated that the absolute impact as measured by excess mortality rates was typically greatest in groups in which the baseline rate of mortality was higher, namely in older age groups and among those with more comorbidities and higher levels of physiologic frailty. However, relative increases in the hazard of mortality during the pandemic were typically greatest among younger age groups and among those with lower physiologic frailty and fewer comorbidities. Patients with dementia had both the highest absolute excess mortality rate and the highest relative increase in mortality. Estimates of excess mortality attenuated but remained elevated after censoring follow-up at first documented SARS-CoV-2 infection or COVID-19, suggesting that factors beyond SARS-CoV-2 infection contributed to the observed excess mortality during the pandemic.

The present analysis adds a unique contribution in the use of individual-level data to estimate and interpret rates of excess mortality associated with the COVID-19 pandemic. Most prior analyses of patterns of excess mortality in the US have used aggregate data.^1,4,5^,^23–25^ In some analyses, deaths have been disaggregated by demographic characteristics, including age, sex, race/ethnicity and region; however, this work has been limited in its ability to adjust for underlying health status. As demonstrated in our previous publication,^26^ estimates of excess mortality from ecological models with aggregate data and survival models with individual-level data yielded nearly identical estimates when adjusting for the same demographic factors. In the present study, we extended our previous report and found that estimates of excess mortality only modestly attenuated from 27% to 25% after additionally accounting for validated, time-updated measures of physiologic frailty and comorbidity burden.

Other distinguishing features of the present study were the availability of verified documentation of laboratory-confirmed SARS-CoV-2 infections, COVID-19 diagnoses and vaccinations at the patient level. We leveraged the available information to censor patients at first evidence of infection, thereby appropriately allowing observed follow-up time without infection to contribute to the analysis. However, this approach is susceptible to the challenges of complete recording of SARS-CoV-2 infections and COVID-19 diagnoses, particularly early in the pandemic before case definitions were standardized and testing was available on a widespread basis. Estimates of excess mortality attenuated from 25% to 19% after implementing this additional censoring, suggesting that factors beyond SARS-CoV-2 infection contributed to the observed excess mortality during the pandemic. In a post-hoc analysis, we observed excess mortality during the first 2 years of the pandemic to be driven by excess risk among patients prior to becoming fully vaccinated. We also observed patterns of excess mortality to reverse after vaccination when compared with unvaccinated pandemic follow-up time—indicating the known protection provided by vaccination—and pre-pandemic follow-up time, which we hypothesize indicates a healthy vaccinee effect.^27^ We suggest interpreting these estimates carefully as some vaccinations delivered outside the VA may not be captured in VA data.

The present study provides a systems-level summary of the overall burden of excess mortality during the pandemic in a national integrated healthcare system. Our findings are consistent with previous reports which demonstrated that the highest excess mortality on an absolute scale was among older patients and those who were more frail or had higher comorbidity burden.^28^ However, these groups were observed to have the lowest excess mortality on a relative scale, likely because the baseline rate of death was already high in these groups and there are many competing causes of death. In addition, our calculations of the number of excess deaths, which incorporates the size of each subgroup, suggested that the largest aggregate burden was among patients aged 65–74 years and those who were least frail or had no recorded comorbidity. Our findings strongly suggest that each of these metrics is important and offers a different story in terms of the impact of COVID-19 on excess mortality in the VA. Studies estimating excess mortality should present findings on both the absolute and relative scales to enable policymakers and operations managers to determine where to allocate resources as we emerge from the pandemic and in future similar outbreaks. The lower relative increases in excess mortality among more frail groups and those with more comorbidities have important implications and suggest that forward mortality displacement might be less of a phenomenon during the post-pandemic period than had been proposed.^29^

Although the analysis of the CCI summary score indicated that those with more comorbidities had higher excess mortality on the absolute scale and lower excess mortality on the relative scale, there were some important clinical subgroups that did not follow this pattern. Notably, patients with dementia had the highest excess mortality on both absolute and relative scales, which was previously identified as an important risk group in other healthcare systems, such as that in the UK.^30^ Interestingly, patients with metastatic cancer, who are likely to be at greater risk of SARS-CoV-2 infection or severe COVID-19,^31^ appeared to have no excess mortality during the first 2 years of the pandemic. These findings likely underscore the importance of distinguishing infection risk from mortality risk once infected. Patients with dementia are more likely to reside in nursing homes, making them more likely to acquire SARS-CoV-2 infection in addition to their higher mortality risk once infected.^32^ Patients with metastatic cancer were identified as an at-risk group and instructed to shelter at home or take great precautions in public spaces, which may have reduced their probability of infection.

Although there are many published reports highlighting racial and ethnic disparities in testing positive for SARS-CoV-2,^14^^,^^33^ we have previously shown that there are no racial or ethnic disparities in the probability of 30-day mortality among those who tested positive in the VA.^13^ Although comprising only 1% of the present study, American Indian/Alaska Native patients experienced the largest absolute and relative increases in mortality during the pandemic, highlighting the need for more focused assessment and evidence-based interventions in partnership with affected racial and ethnic minority communities.

This study elucidated patterns of excess mortality associated with the COVID-19 pandemic leveraging individual-level data on demographics and clinical characteristics from a national healthcare system. Study strengths included the ability to adjust estimates of excess mortality for underlying health status and compare the magnitude of excess mortality within levels of physiologic frailty and comorbidity burden. This study also has some limitations. First, this study included Veterans currently receiving care in the VA, who are older and have a higher prevalence of chronic health conditions than the general US population.^34^^,^^35^ Prior research has established that after adjusting for age, sex, race/ethnicity, region and rural/urban residence, all of which were included in this study, there is no difference in the total disease burden between Veterans and non-Veterans.^36^ Although we expect absolute measures to differ across the population under study, relative measures in the present study are more likely to generalize to the general US population, which we have demonstrated in previous work.^26^ Second, whereas most variables used in the present study were complete, nearly one in four patients had missing labs to calculate the VACS Index and were categorized separately. In addition, our measure of comorbidity burden was based on the presence or absence of diagnostic codes. Any incompleteness in the recording of diagnoses would bias the CCI score downwards, which could increase the potential for inflated estimates of excess mortality given that individual-level comorbidity is likely to increase as the individual ages from the pre-pandemic to pandemic periods. Together, although the present study builds upon previous work through the addition of individual-level measures of the CCI and VACS Index, there may be residual confounding in the reported estimates. Third, although secondary analyses utilized records of laboratory-confirmed SARS-CoV-2 infections and COVID-19 diagnoses from both VA and external sources, some patients may be misclassified if they tested positive elsewhere and did not self-report the diagnoses at a subsequent VA visit. The US Centers for Disease Control and Prevention (CDC) nationwide seroprevalence study estimated that cumulative reported COVID-19 cases were 14.9% and infection-induced seroprevalence was 28.8% in December 2021.^37^^,^^38^ Among patients in the present study, 11.4% had a record of a SARS-CoV-2 infection or COVID-19 diagnosis by February 2022. Patients with less severe infections identified through home testing as well as those who died from severe infections in non-VA hospitals are both likely to be misclassified, which would influence our results of the secondary analyses in both directions.

In conclusion, we report important differences in patterns of excess mortality between clinically defined risk groups. The relative increase in mortality was smaller in groups with higher frailty and comorbidity burdens. However, because baseline death rates were higher in those groups, the absolute increase in mortality rates was greatest among these higher-risk groups. The use of individual-level data provided important clinical context for patterns of excess mortality in the USA during the COVID-19 pandemic.

## Ethics approval

This study was approved by the institutional review boards of Yale University (ref. #1506016006) and VA Connecticut Healthcare System (ref. #AJ0013). It has been granted a waiver of informed consent and is compliant with the Health Insurance Portability and Accountability Act.

## Supplementary Material

dyad136_Supplementary_Data

## Data Availability

Due to US Department of Veterans Affairs (VA) regulations and our ethics agreements, the analytic data sets used for this study are not permitted to leave the VA firewall without a data use agreement. This limitation is consistent with other studies based on VA data. However, VA data are made freely available to researchers with an approved VA study protocol. For more information, please visit https://www.virec.research.va.gov or contact the VA Information Resource Center at VIReC@va.gov.
